# Supplementary material for: Cation Vacancies in Feroxyhyte Nanosheets toward Fast Kinetics in Lithium–Sulfur Batteries
Source: Nanomaterials (Basel). 2023 Feb 28;13(5):909. doi: 10.3390/nano13050909 (PMC10005701; doi:10.3390/nano13050909)
Supplement: Supplementary file 1 [file nanomaterials-13-00909-s001.zip › nanomaterials-2236279-supplementary.pdf]

Supplementary Materials

Cation Vacancies in Feroxyhyte Nanosheets toward Fast Kinetics in Lithium–Sulfur Batteries

Aimin Niu, Jinglin Mu, Jin Zhou, Xiaonan Tang*, and Shuping Zhuo*

School of Chemistry and Chemical Engineering, Shandong University of Technology, Zibo 255049, China
* Correspondence: xntang@sdut.edu.cn (X.T.); zhuosp_academic@yahoo.com (S.Z.)

Experimental section

1. Material synthesis

1.1. Synthesis of rGO/CNT

Firstly, graphene oxide (GO) was synthesized from natural graphite powder using a modified Hummers method [1]. CNT was purchased by Sinopharm Chemical Reagent Co., Ltd. Afterwards, the acidifying CNT was added into the above solution by the mass ratio of 1:1 with probe sonication for another 30 min. In order to obtain rGO/CNT, the GO/CNT by freeze drying was transferred to the tube furnace and heated to 800 °C at a rate of 5 °C min^{-1} in Argon and kept at this temperature for another 2 h.

1.2. Preparation of v-FeOOH /rGO/CNT@S cathode

In a typical procedure, CTAB (5 mmol) was dissolved in 50 mL of rGO/CNT solution in a 100 mL round-bottomed flask followed by purging with N_2 about 15 min. $FeSO_4{\cdot}7H_2O$ (1.5 mmol) was added into the flask. Afterward, a freshly prepared sodium borohydride solution (1.5 mmol, 2 mL) was added dropwise. With the flow of N_2, iron salt reacted with sodium borohydride to produce black iron-boron composite comprising iron crystallites embedded within an amorphous boron nanosphere matrix, which gradually grew on the rGO/CNT. After 40 min, N_2 was turned off and the sample was exposed to air for 4 h. After being washed with distilled water and ethanol several times, the v-FeOOH /rGO/CNT was obtained. The preparation of v-FeOOH/rGO/CNTs@S cathode materials were prepared through a typical melt-diffusion approach. In detail, S was mixed with the host materials with a weight ratio of 60:40. Then the mixture was transferred to a Ar filled glass bottle and heated at 155 °C for 12 h.

1.3. Preparation of FeOOH/rGO/CNT@S cathode:

As a control sample, FeOOH without Fe vacancies (named FeOOH) based on rGO/CNT was prepared by rapid oxidation of $Fe(OH)_2$ particles with H_2O_2. Specifically, 1.5mmol of $FeSO_4{\cdot}H_2O$ were dissolved in 40 mL of rGO/CNT solution,10 mL of NaOH (10 M) was added to the above solution at 45 °C in N_2 atmosphere. The resulting $Fe(OH)_2$ film on rGO/CNT was aged at 45 °C for 6 h, and then5 mL of H_2O_2 (30%) solution was instantly poured into the suspension and a further 10 mL of the H_2O_2 solution was added. The resulting products was taken out and washed with distilled water and ethanol several times, and dried. The prepared of FeOOH /rGO/CNT@S cathode is same as above.

1.4. Lithium polysulfide (Li_2S_6) solution

Li_2S and sulfur with a molar ratio of 1:5 were added into a DOL/ DME mixture (1:1, v/v) and stirred overnight at 60 °C. The concentration of the Li_2S_6 solution was controlled to be 0.2 mol L^{-1}.

1.5. Li_2S nucleation tests

Li_2S_8 solution as catholyte was first prepared by mixing sulfur and lithium sulfide with a molar ratio of 7:1 in a tetraglyme solvent, followed by vigorous stirring for 24 h. The concentration of Li_2S_8 was 2.0 mol L^{-1} [S].

The corresponding electrodes were fabricated without sulfur. The active material (v-FeOOH , FeOOH) and poly(vinylidene fluoride) (PVDF) with the mass ratio of 9:1 were dispersed in N-methyl pyrrolidone (NMP) solusion under virgrous stiring to form uniform slurry, which was subsequently coated on Al foils. Typically, the v-FeOOH or FeOOH acted as the cathodes and lithium foil served as the anode. Celgard 2400 membrane as the separator was inserted into compartment of between cathode and anode. 20 μL Li_2S_8 catholyte was added into the cathode and 20 μL LiTFSI (1.0 mol L^{-1}) without Li_2S_8 was dropped onto the anode compartment. The assembled batteries were galvanostatically discharged at 0.112 mA to 2.06 V and then kept potentiostatically at 2.05 V till the current was below 10^{-5} A. The entire process was analyzed to evaluate the nucleation rate of Li_2S according to Faraday's law.

1.6. Density Functional Theory Calculations

The density functional theory (DFT) calculations were performed with $Dmol^3$ implemented in the Materials Studio 2019, using Perdew-Burke-Ernzerhof (PBE) generalized gradient approximation, double numeric polarized (DNP) basis set and D2 dispersion correction. The FeOOH (001) surface was chosen for the calculation. The Fe-deficient FeOOH (001) surface was built by removing Fe, O and H atoms from intact FeOOH surface. A 2 × 2 supercell was used and the vacuum height was 15 Å. Convergence tests of Monkhorst-Pack grids were made to ensure the results. The adsorption energies (Eads) of Li_2S_6 on FeOOH (001) and Fe-deficient FeOOH (001) surface were calculated.

2. Characterization and electrochemical measurements

2.1. Electrochemical Characterization

The cathode slurry was prepared by mixing 80 wt% S-FeOOH with FeVs, 10 wt% PVDF binder, and 10 wt% super p in 1-methyl-2-pyrrolidinone (NMP) dispersant solvent, and the mass density of sulfur was controlled to be 0.5 mg cm^{-2}.The slurry was cast on carbon coated aluminum foil and dried at 60 °C for 12 h. Bis-(trifluoromethanesulfonly) imide (LiTFSI, 1.0M) with $LiNO_3$ (0.1M) dissolved in 1,3-dioxolane (DOL) and 1,2-dimethoxyethane (DME) (v/v, 1:1) was used as electrolyte.CR2032 coin cells were assembled with S-FeOOH with FeVs(S-FeOOH without FeVs)cathode, polypropylene separator (Celgard) and lithium foil anode in an argon-filled glove-box. The charge-discharge cycling was carried on LAND battery test station within the voltage range of 1.5 to 3.0 V versus Li^+/Li. Versa STAT4 (Princeton Applied Research) was used to test cyclic voltammetry (CV) at different scanning rate and electrochemical impedance spectroscopy (EIS) was conducted by Modulab from 0.01 kHz to 1000 Hz.

2.2. Symmetrical cell assembly and measurements

The FeOOH with FeVs (FeOOH without FeVs) composite was mixed with the PVDF binder with a weight ratio of 90:10. Then the slurry was coated onto the Al foil and dried at 60 °C for 12 h in a vacuum oven. The electrode was punched into a circular shape with diameter of 1.2 cm. The mass loadings of both FeOOH with FeVs and FeOOH without FeVs electrodes are around 0.6mg cm^{-2}. The electrodes were used as identical working and counter electrodes, and 40 μL electrolyte (in DOL/DME, v/v = 1:1) containing 0.2 M Li_2S_6 and 1 M bis(triflfluoroethanesulfony)imide lithium (LiTFSI) was added into each cell. Cyclic voltammetry (CV) measurements were carried out using an electrochemical workstation (VMP3, Bio-Logic, Claix, France) at 0.5, 20 ,50 mVs^{-1} with a voltage window between −0.8 and 0.8 V.

2.3. Material characterization

The morphologies and microstructures of as-prepared samples are examined by a scanning electron microscope (SEM, Tescan Mira 3) and a transmission electronic microscope (Talos F200S). The crystal and phase of the compositions are performed on powder X-ray diffraction (XRD, Rigaku-mini Flex600). The surface electronic states were analyzed by X-ray photoelectron spectroscopy (XPS, Thermo Scientific K-Alpha) using monochromatic Al Kα X-rays. In situ XRD measurement was performed in a steel Swagelok-type cell. The cell was connected to a multichannel battery testing system (Neware, Shenzhen, China) and the XRD patterns were collected at a Bruker D8 advance X-ray diffractometer with Cu Ka (λ = 0.154 nm) radiation.

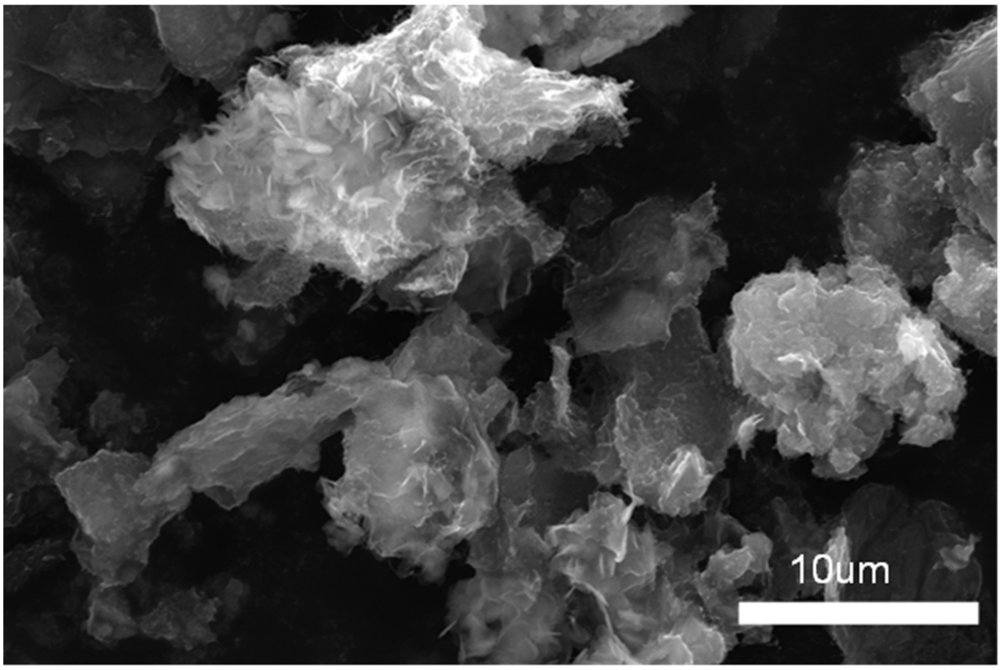

Figure S1. SEM of v-FeOOH /rGO/CNT.

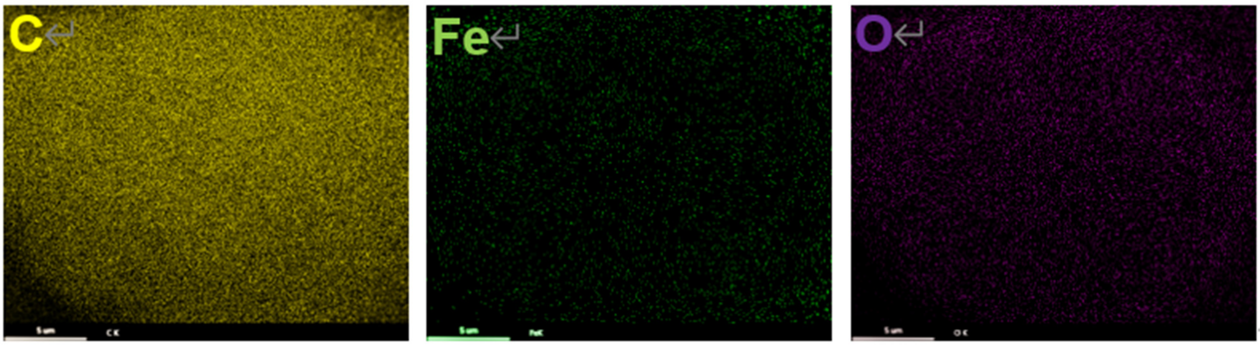

Figure S2. EDS elemental maps of C (yellow), Fe (green) and O(purple) of v-FeOOH /rGO/CNT.

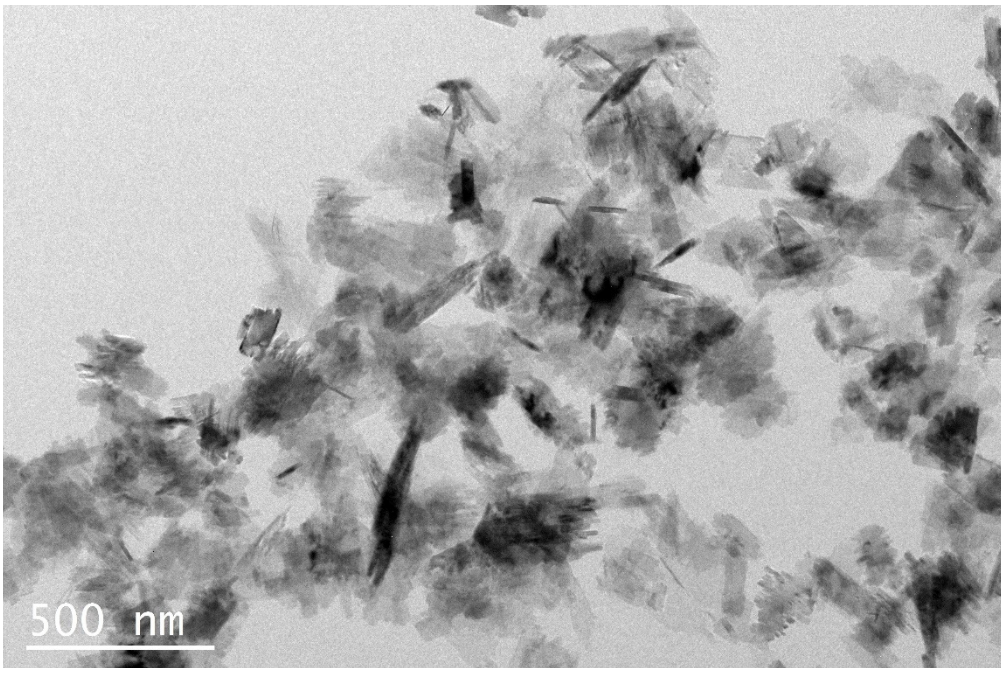

Figure S3. TEM of v-FeOOH.

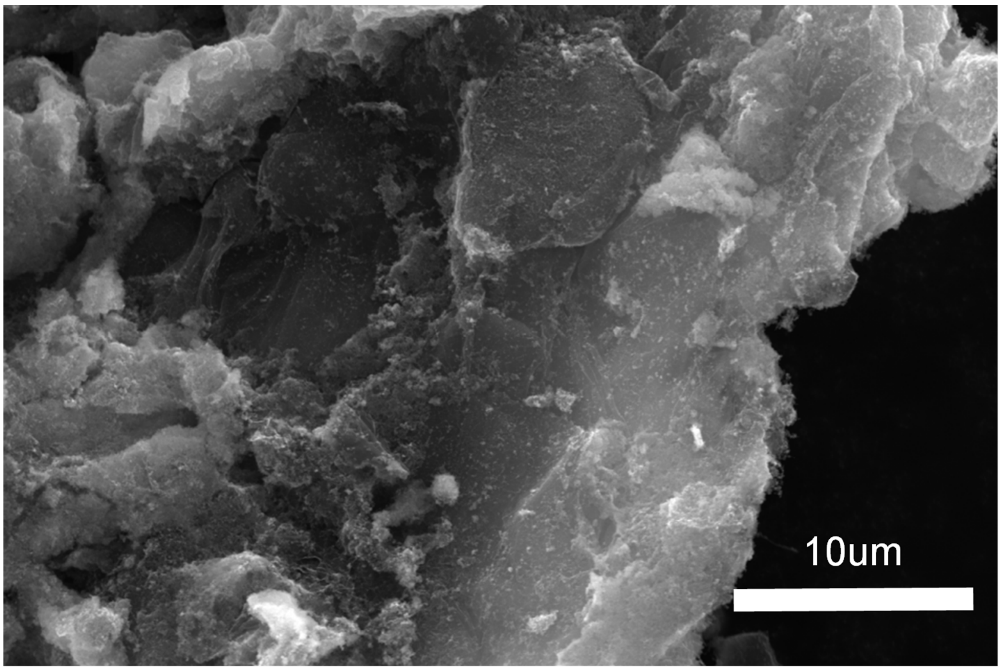

Figure S4. SEM of FeOOH /rGO/CNT.

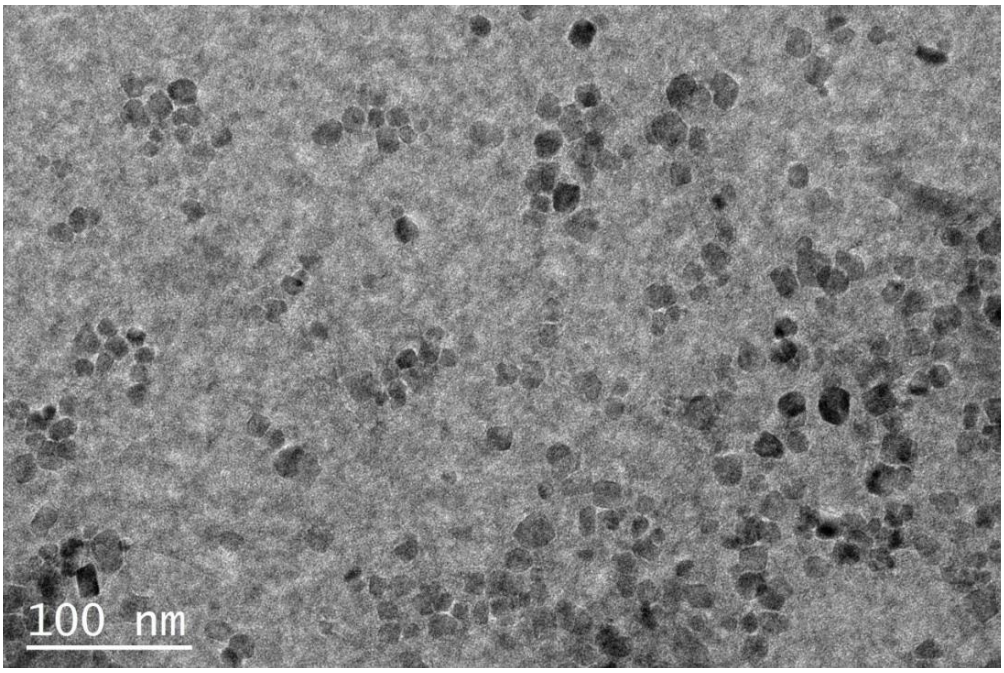

Figure S5. TEM of FeOOH.

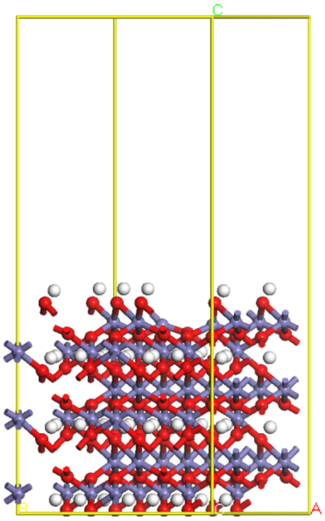

Figure S6. The side view of the optimized FeOOH structure.

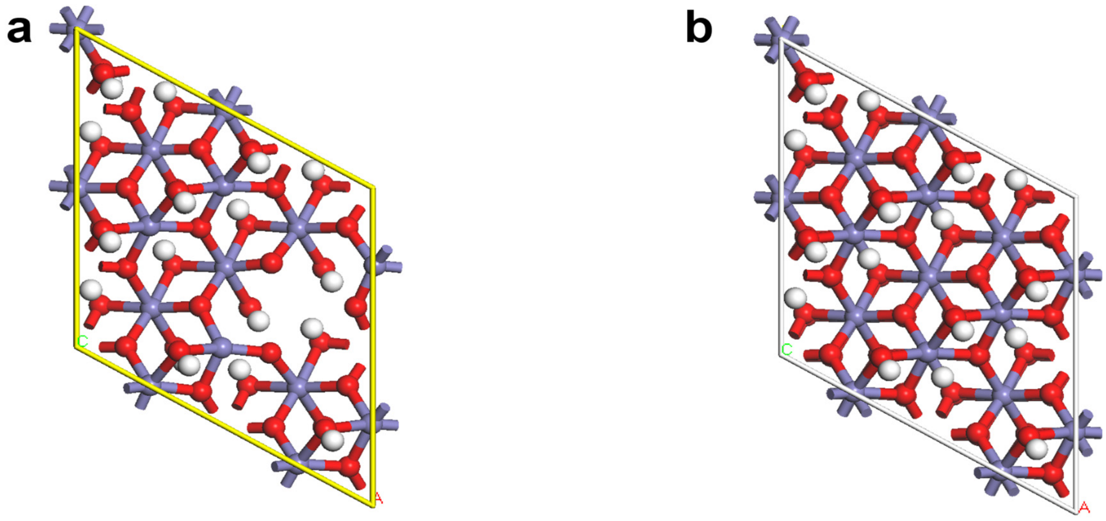

Figure S7. The planform of the optimized FeOOH with FeVs and FeOOH without FeVs structure.

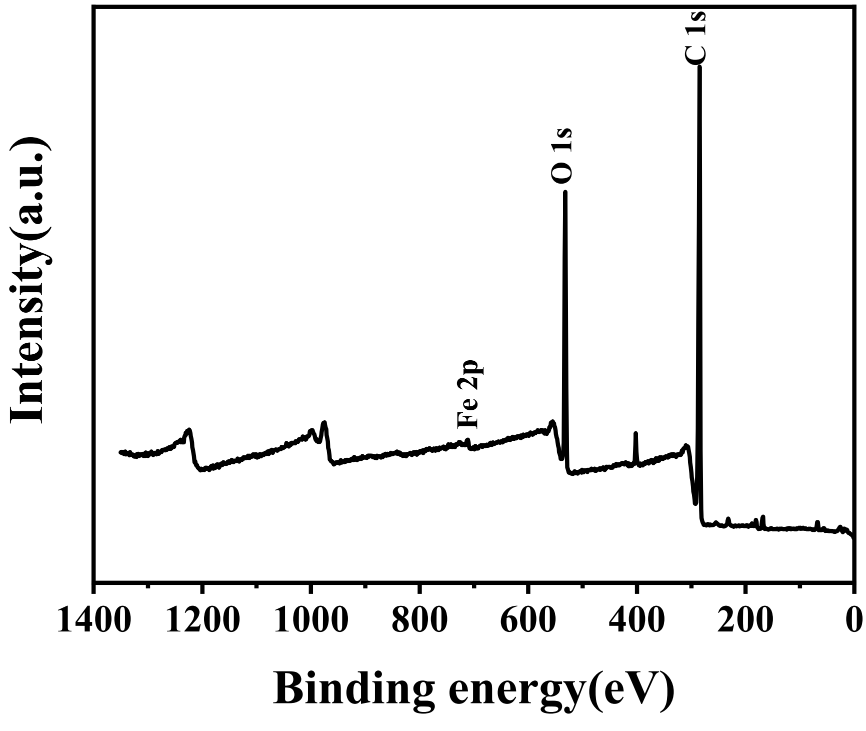

Figure S8. The XPS survey spectrum of v-FeOOH

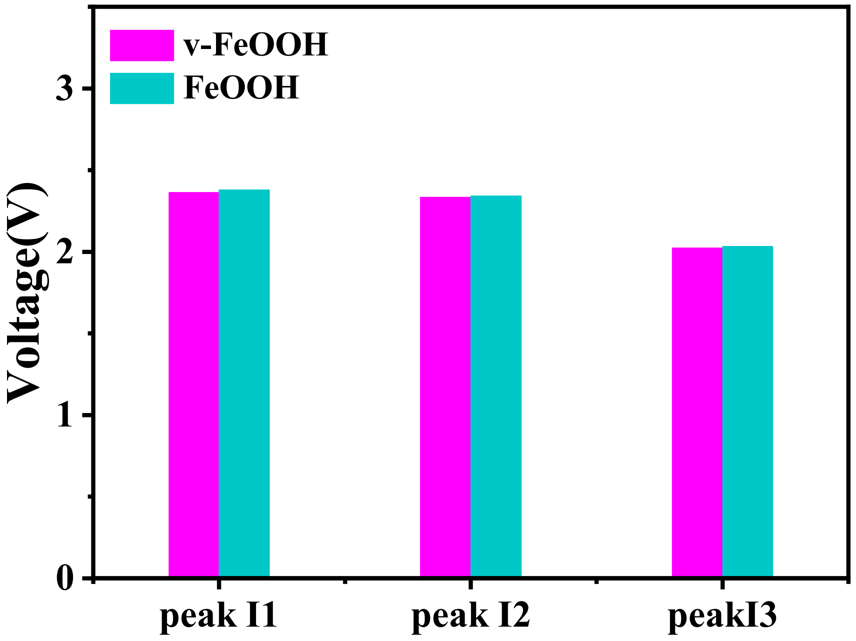

Figure S9. The peak potentials of v-FeOOH and FeOOH at the scan rate of 0.2 mV s^{-1}.

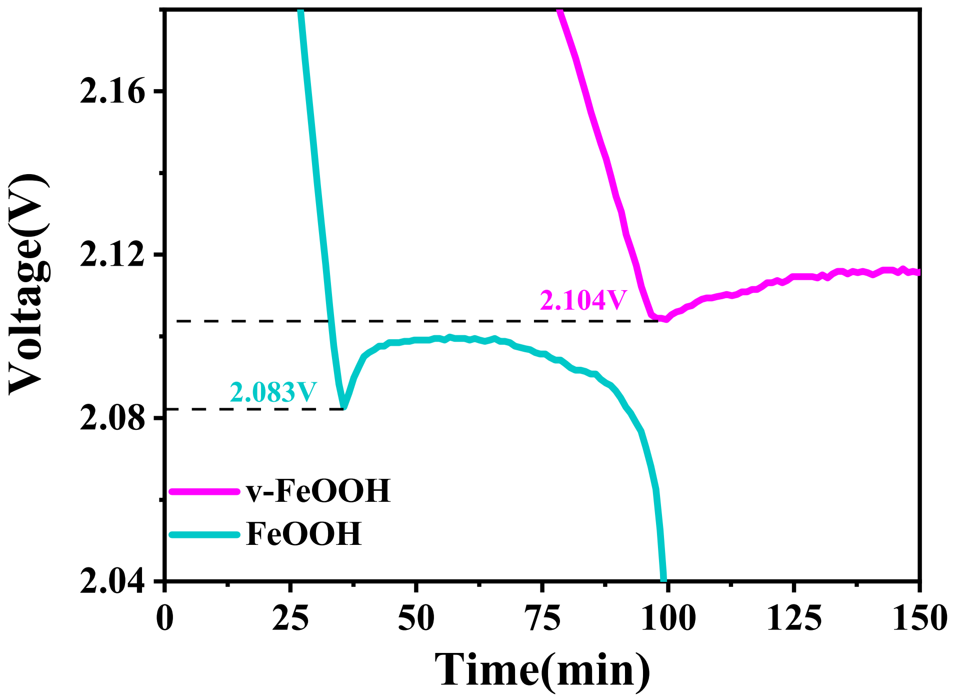

Figure S10. The discharge overpotential of v-FeOOH and FeOOH.

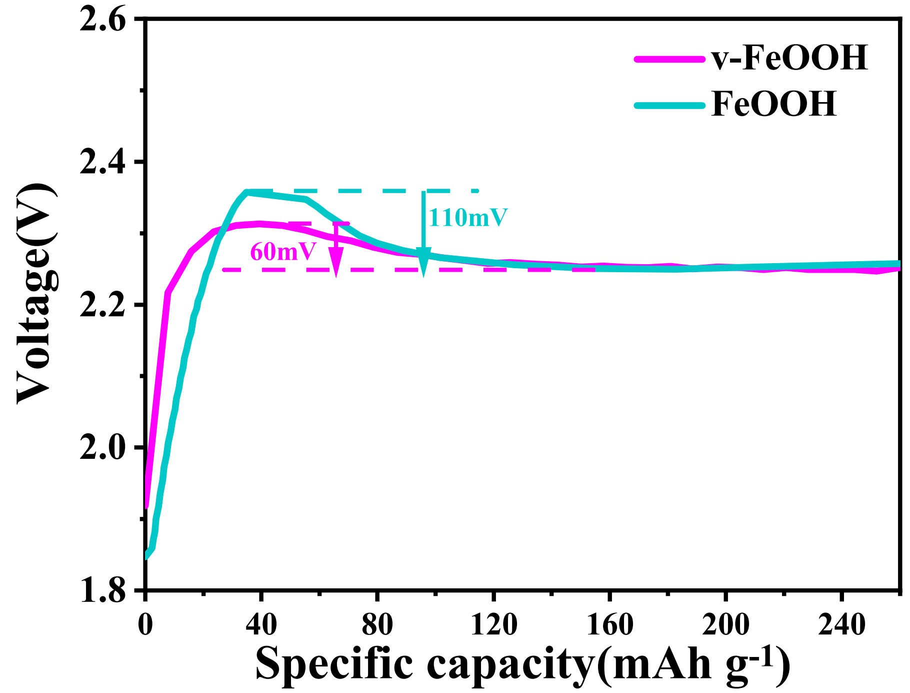

Figure S11. Charge voltage profiles of v-FeOOH and FeOOH cells at 1C.

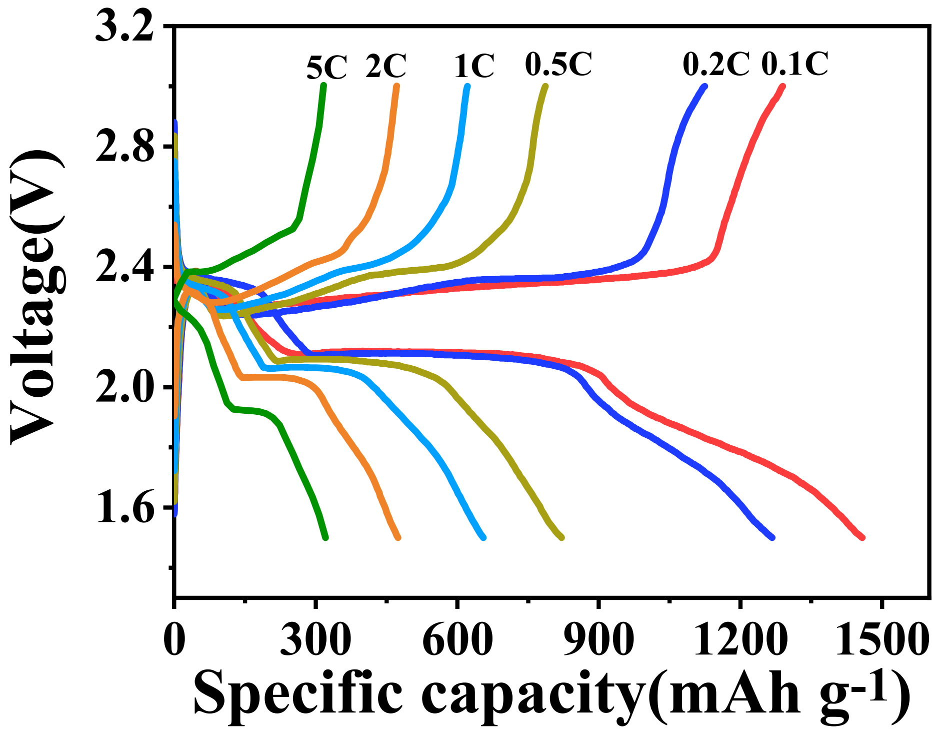

Figure S12. Charge-discharge profiles of the v-FeOOH at different current rates.

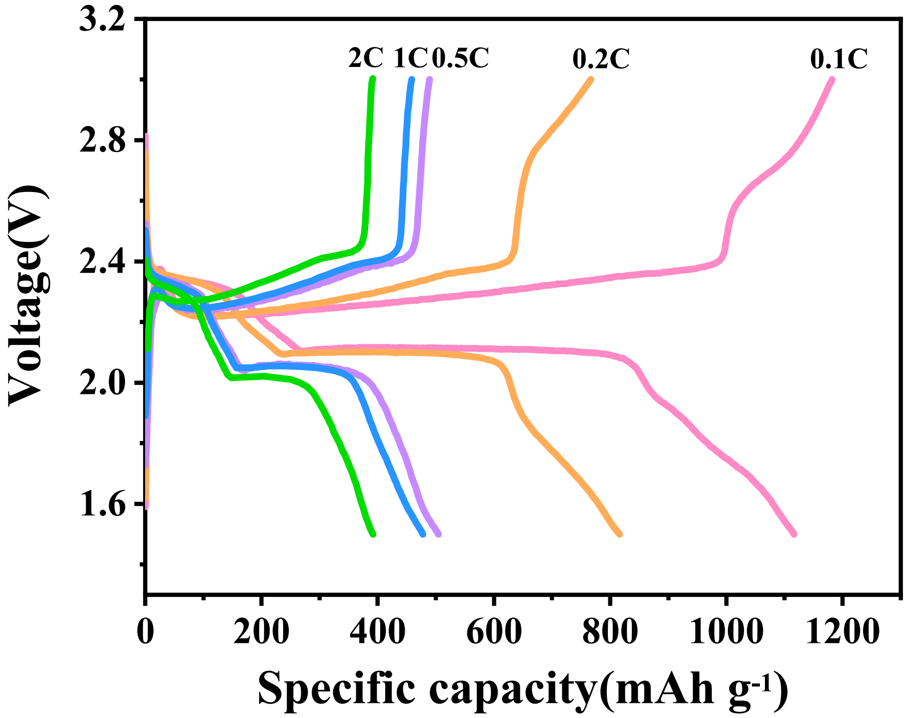

Figure S13. Charge-discharge profiles of the FeOOH at different current rates.

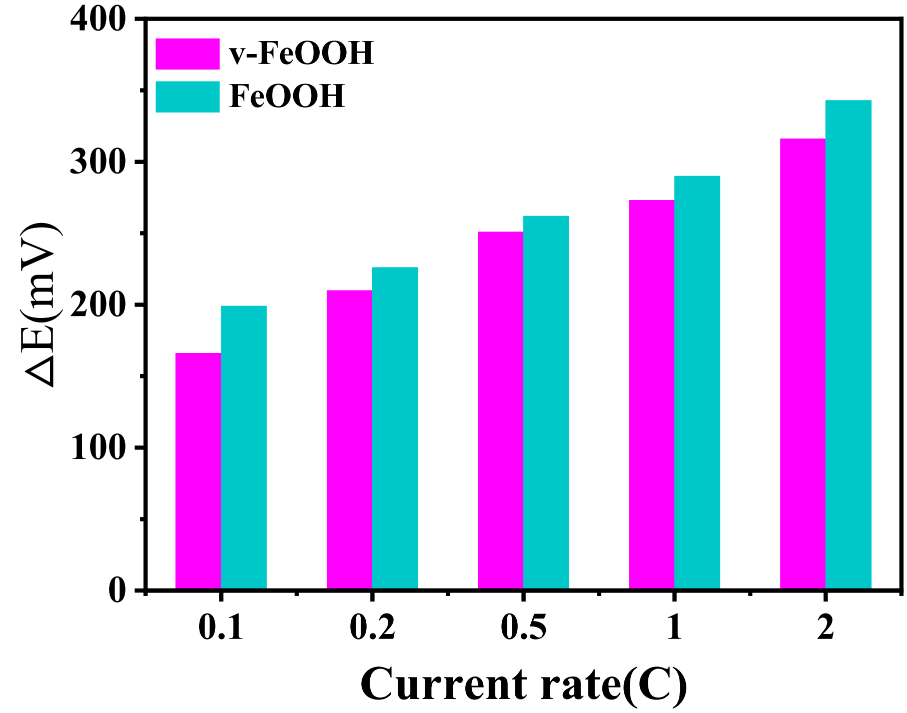

Figure S14. Overpotentials (ΔE) of the v-FeOOH and FeOOH at different current rates.

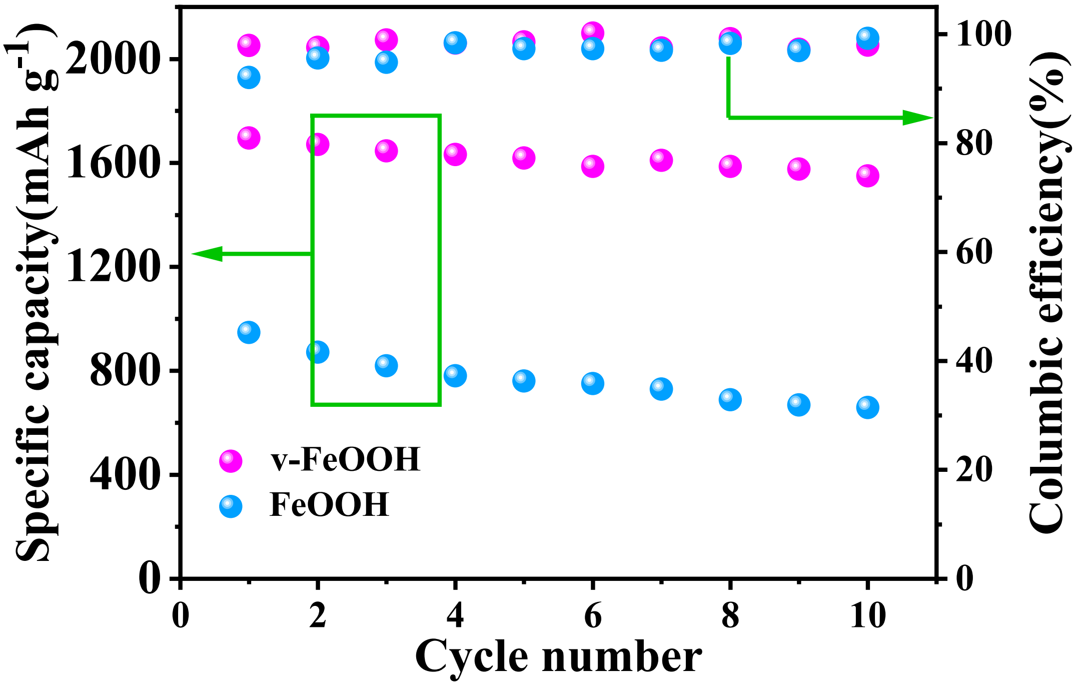

Figure S15. Prolonged cycling stability at 1 C of v-FeOOH and FeOOH electrodes of the first ten cycle.

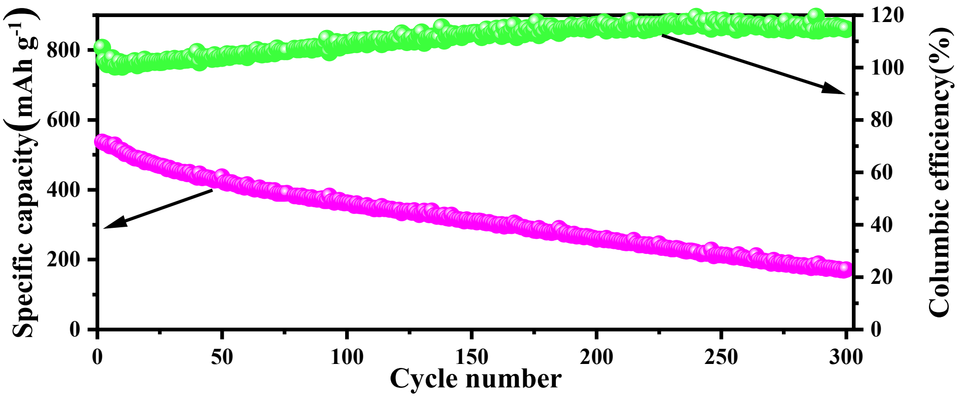

Figure S16. The cycling performance of v-FeOOH at 5C over 300 charge-discharge cycles.

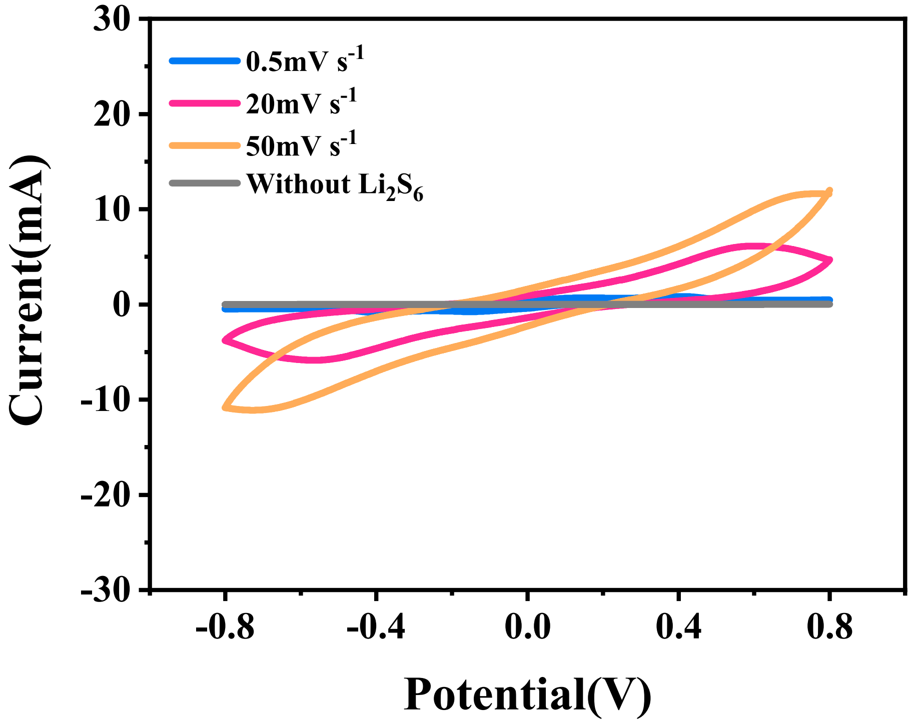

Figure S17. CV of Li_2S_6 and Li_2S_6-free symmetrical cell with FeOOH working electrode at different scan rate.

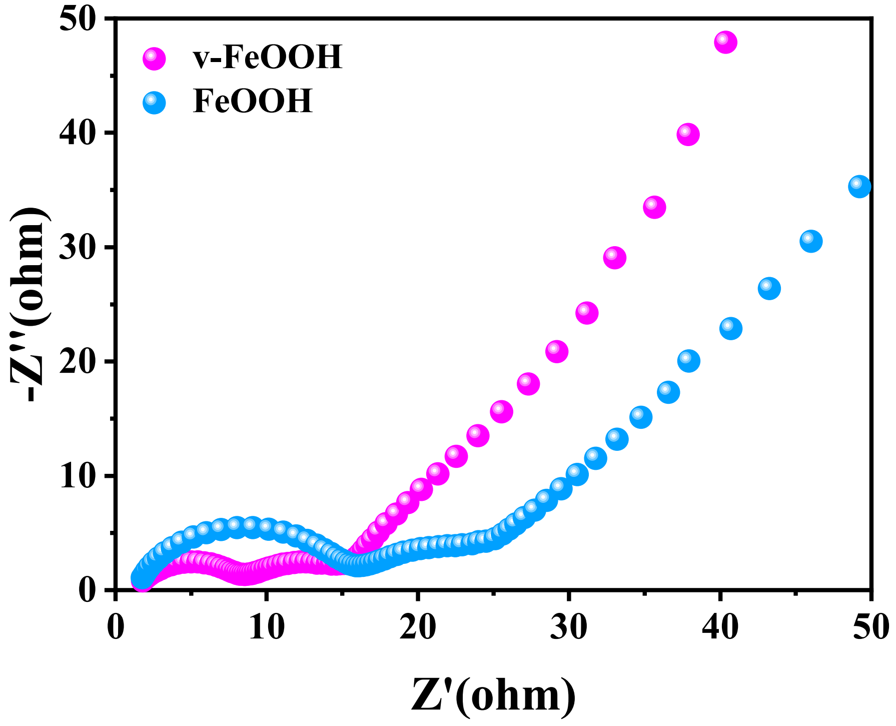

Figure S18. EIS of the Li_2S_6 symmetrical cell with v-FeOOH and FeOOH working electrodes.

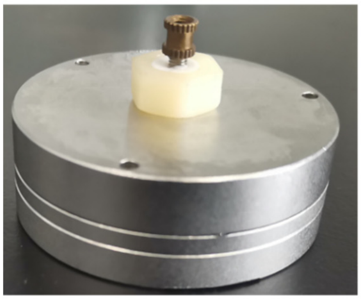

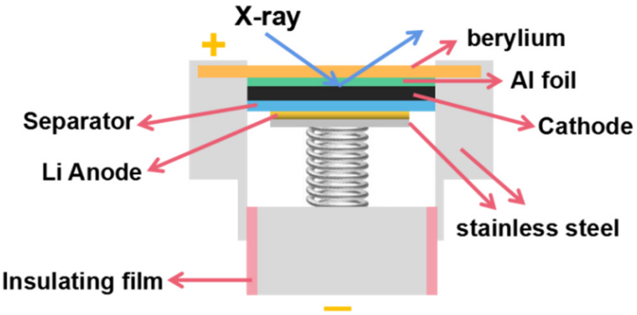

Figure S19. Left: photograph of the *in situ* XRD setup. Right: schematic of the cross section of Li-S battery in a *in situ* XRD cell.

Reference:

1. Marcano, D.C.; Kosynkin, D.V.; Berlin, J.M.; Sinitskii, A.; Sun, Z.; Slesarev, A.; Alemany, L.B.; Lu, W.; Tour, J.M. Improved synthesis of graphene oxide. *ACS Nano* **2010**, *4*, 4806–4814. https://doi.org/10.1021/nn1006368.
